# A Prototype SSVEP Based Real Time BCI Gaming System

**DOI:** 10.1155/2016/3861425

**Published:** 2016-03-09

**Authors:** Ignas Martišius, Robertas Damaševičius

**Affiliations:** ^1^Department of Computer Science, Kaunas University of Technology, Studentu 50-415, LT-51368 Kaunas, Lithuania; ^2^Department of Software Engineering, Kaunas University of Technology, Studentu 50-415, LT-51368 Kaunas, Lithuania

## Abstract

Although brain-computer interface technology is mainly designed with disabled people in mind, it can also be beneficial to healthy subjects, for example, in gaming or virtual reality systems. In this paper we discuss the typical architecture, paradigms, requirements, and limitations of electroencephalogram-based gaming systems. We have developed a prototype three-class brain-computer interface system, based on the steady state visually evoked potentials paradigm and the Emotiv EPOC headset. An online target shooting game, implemented in the OpenViBE environment, has been used for user feedback. The system utilizes wave atom transform for feature extraction, achieving an average accuracy of 78.2% using linear discriminant analysis classifier, 79.3% using support vector machine classifier with a linear kernel, and 80.5% using a support vector machine classifier with a radial basis function kernel.

## 1. Introduction

Since the first experiments of electroencephalography (EEG) on humans in 1929, the EEG of the human brain has been used mainly to evaluate neurological disorders in the clinical environment and to investigate brain functions in the laboratory. An idea that brain activity could be used as a communication channel has gradually emerged. The possibility of recognizing a single message or command considering the complexity, distortion, and variability of brain signals appeared to be extremely remote. Yet EEG demonstrates direct correlations with user intentions, thereby enabling a direct brain-computer interface (BCI) communication channel. BCI requires high computational capacity to analyse brain signals in detail and in real time, and until recently the requisite technology either did not exist or was extremely expensive. The continuing development of computer hardware and software now supports highly sophisticated online analysis of many signal channels at high speed. Also, greatly increased social recognition of the needs and potential contributions of people with severe neuromuscular disorders such as spinal cord injury has generated clinical, scientific, and commercial interest in better communication and control technology. An interdisciplinary field of research has been created to offer direct human-computer interaction via signals, generated by the brain itself.

Brain-computer interface (BCI) technology is a communication channel that enables users to control devices and applications without the direct use of muscles [1]. The development of cognitive neuroscience field has been instigated by recent advances in brain imaging technologies such as electroencephalography, magnetoencephalography, and functional magnetic resonance imaging. The growing field of BCI research is relatively new. The first BCI prototype was created by Dr. Vidal in 1973 [2]. This system was intended to be used as a promising communication channel for persons with severe disabilities, such as paralysis, amyotrophic lateral sclerosis, brain stroke, or cerebral paralysis [3]. Continuation and acceleration of recent progress in BCI research and development have begun to address real world applications spanning activities of daily living, environment control, exercise, locomotion, and verbal communication [4].

The BCI technology, combined with ambient assisted living (AAL) systems, can potentially make the home environment more intelligent and assistive, providing alternative communication means for supporting independent life of elderly people affected by impairments. The quality of life of persons suffering from severe motor disabilities can benefit from the use of BCI-based assistive technology [5]. Despite recent developments, there are still numerous obstacles to building a usable and effective BCI system. The biggest challenges are related to accuracy, speed, price, and usability. Current BCI systems are inaccurate and have a low information transfer rate. This means that the user needs a long period of time in order to send commands to the device that is being controlled. Another problem is the high cost of EEG equipment, such as an EEG cap and amplifiers [6]. Systems with a high sensor count take a long time to prepare for use and are uncomfortable. Due to these limitations, no BCI system has become commercially successful to this date. Sound knowledge of the data acquisition process, EEG waveform characteristics, signal processing methodologies for feature extraction, and classification is a prerequisite before attempting to design and implement a functional BCI system. These research points have been highlighted by the BCI development community as being both important and necessary, for further BCI development [7–9].

Therefore, BCI technology still has many problems to be solved to transit to feasible assisted living [10] with minimal training effort and support required for independent use at home. One approach is to develop BCI applications based on a user centred design approach to bridge the gap between BCI systems and their end users [11]. Another approach is gamification, that is, the use of elements of a game in a serious nongame context [12]. Redefinition of daily control tasks as enjoyable multimedia applications could define a new level of control possibilities for the disabled but also for healthy users [13].

The goal of this paper is to explore the BCI technology as a gaming controller option, which can require less EEG quality and present low risk interactions. By using low cost devices such as the Emotiv EPOC headset, aimed at consumers rather than scientists and medics, in the system, we sacrifice performance for price and comfort of the system user. Researchers have already applied the Emotiv neuroheadset's technology in a variety of ways: Liu et al. [14] compared the EPOC device to a g.USBamp device in a steady state visually evoked potential (SSVEP) system with good results. It is also used in other paradigms, such as the P300-based system, developed by Duvinage et al. [15]. In this paper we use and compare linear discriminant analysis (LDA) and support vector machine (SVM) classifiers with brainwave data features obtained using wave atom transform (WAT) for the control of a prototype SSVEP based BCI game.

The structure of the remaining parts of the paper is as follows.  Section 2 discusses applications of BCI technology.  Section 3 describes the typical architecture of BCI systems.  Section 4 discusses the more commonly used BCI paradigms.  Section 6 describes the materials and methods used.  Section 7 presents the experimental results. Finally, Section 8 presents conclusions.

## 2. Applications of BCI Technology

BCI design represents a new frontier in science and technology that requires multidisciplinary skills from fields such as neuroscience, engineering, computer science, psychology, and clinical rehabilitation. BCI research has been successfully used not only for helping the disabled [16], but also as being an additional data input channel for healthy people. It can be exploited as an extra channel in game control [17], augmented reality applications [18], household device control [19], fatigue and stress monitoring [20], and many other applications.

The applications of BCI can be divided into two main categories, medical applications and nonmedical applications, such as multimedia or virtual reality. The first category includes the following:

- *Rehabilitation and Prosthetic Device Control*. The BCI technology is used for patients with moderate to severe movement disabilities. Although rehabilitation is impossible in some diseases, such as amyotrophic lateral sclerosis, some of the patients, that is, stroke patients, can sometimes regain some or all lost motor control with effective rehabilitation. Motor imagery (MI) BCI can be used as a means for rehabilitation. In studies [21, 22] among others, patients have tried to grasp objects using BCI controlled robotic prosthetic hands. Robotic arms provided feedback for the patients, aiding their rehabilitation. While rehabilitation results show potential, robotic prosthetic limb control requires a number of control commands, not achievable by BCI systems. The experiments, therefore, are mostly limited to the 1D or 2D movement control.
- *Medical Diagnosis*. BCI technology can be used for developing health monitoring applications that may periodically screen the user for early indicators of neural diseases such as epilepsy [23] and suggest the user to see a doctor for diagnosis.
- *Assistive Mobility*. The most beneficial devices for disabled people are those that let them regain mobility. This is achieved by providing wheelchair control, by means of BCI. BCI-driven spelling devices are used to spell letters or words, allowing for disabled communication. The P300 speller is one of the most famous BCI paradigms [24].
- *BCI Controlled Web and Music Browsers*. Internet access has become the main source of communication on a global scale. The BCI technology enables the development, to make the internet accessible for the disabled. As more aspects of daily life become accessible online (education, retail, personal finance, or business), the potential benefit of connectivity also increases. In a study [25], patients used the P300 paradigm to navigate text, browse forward and backward, use bookmarks, and spell text.
- *Mental State Recognition.* Work in this area deals with the recognition of mental states, such as attention levels, to treat attention deficit disorder patients [26], workload, and fatigue [27], useful for an operators cognitive state assessment.

Although the BCI technology is mainly designed with disabled people in mind, it can also be beneficial to healthy subjects. EEG is particularly suited for this purpose, because it is noninvasive, portable, has a good temporal resolution of a few milliseconds, and is relatively low cost. Therefore the nonmedical applications of BCI include the following:

- *Gaming*. All BCI paradigms have been exploited for gaming purposes. BCI is used either as a primary means to control the game or as an extra channel for in game communication, to perform certain user actions, whereas the game is primarily controlled by traditional means. The game examples include a 3-class motor imagery-based asteroid-dodging game, described in [28], and a BCI control interface for a popular game “World of Warcraft” [29]. The SSVEP based games include a 2-class game called MindBalance [30], P300-based MindGame [31] as well as Pinball [32], Pacman [33], and Tetris [34].
- *Virtual Reality*. Most existing works focus on either rotating the virtual camera or traveling in the virtual environment. Pineda et al. used a BCI based on the mu rhythm to interact with a “First Person Shooter” video game [35]. A high mu rhythm level triggered left camera rotation, whereas low mu levels triggered right rotations. Other commands in the game were issued by using the keyboard.

## 3. Typical Architecture of BCI Systems

A BCI is an artificial intelligence system that can recognize patterns in brainwaves in these stages: signal acquisition, preprocessing or signal enhancement, feature extraction, classification, and the control interface [36]. Designing a BCI system is a multidisciplinary task, involving knowledge and methods adopted from the areas of computer science, signal processing, neurology, and physiology.

To use a BCI, two stages are required: (1) a training stage, in which (a) the user is trained to willingly control his brain potentials (in the case of operating condition BCI), (b) an offline training stage, which calibrates the training algorithm (in the case of pattern recognition BCI), and (2) the online stage, in which the BCI system is used for control.

**Figure 1:**
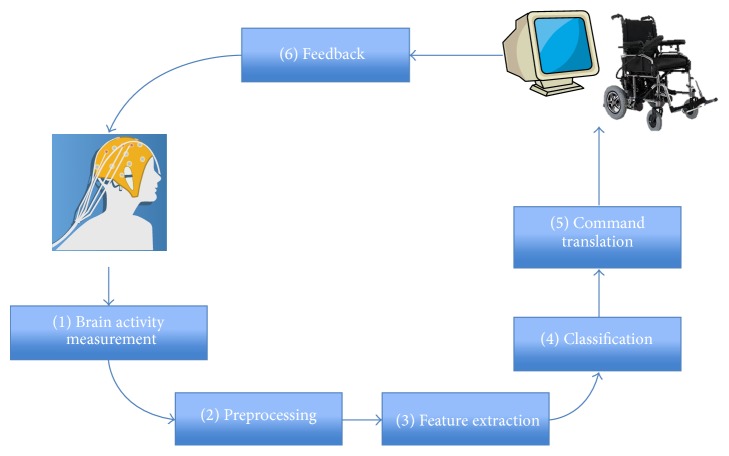
General architecture of an online BCI.

In the online mode, the BCI system generally performs a six-step process (see Figure 1): brain activity measurement, preprocessing, feature extraction, classification, command translation, and feedback [37].

1. Brain activity measurement is the step in which electrodes are used to obtain the user's EEG at specific regions on the scalp, to form input for the BCI system. This step involves determining the number and location of the channels, amplification, analogue filtering, and A/D conversion. Channel locations are selected according to the paradigm used and mental task performed.
2. The preprocessing step consists of denoising the recorded brain signal in order to enhance the relevant information inside. Denoising can be performed by channel or artefact rejection, DSP signal filtering methods. Preprocessing involves the preparation of the EEG recordings. It is an important stage that decides the filtering, segmentation, and detrending methods used to prepare the EEG data for further stages. Filtering and segmentation (also known as epoching) are used to identify and maximize the information over a certain time or frequency range that is associated with the characteristic brain activity to be elicited. Most cognitive EEG activity is usually in the range of 0.2–40 Hz; thus filtering outside of this range reduces noise. A band-pass filter at the electrical mains frequency is typically performed in addition. After filtering, the segmentation of EEG data is performed. This involves splitting the continuous EEG signal into time-locked windows, which usually overlap or are locked to a stimulus (in case of synchronous BCIs). Epoching allows for averaging and dramatically simplifies the feature extraction and classification process. Detrending removes any baseline drift associated with the EEG recordings. This is important to ensure the quasi-stationarity of small EEG segments. The sample rate can be converted to represent the data in as few samples as possible to reduce the computational demands of processing a large number of samples. The sampling rate must be chosen to be at least twice that of the maximum frequency contained in the data (Nyquist rate [38]). A sampling rate of 128 Hz can record frequencies up to 64 Hz, thus capturing the entire range of EEG waves.
3. Feature extraction is a step to describe the signal by a few relevant, command-related values known as “features.” This stage often characterizes the BCI design approach. Features that describe the signal in as few components as possible are resilient to noise and artefacts have to be identified and used. Identifying and extracting good features from signals is a crucial step in the design of BCI systems. If the features extracted from EEG are not relevant and do not describe the signal well enough, the classification algorithm which will use these features will have trouble classifying the mental states of the user; the correct recognition rates of mental states will be low, in which case the use of the interface would be impossible or inconvenient. Thus, even if it is sometimes possible to use raw signals as the input of the classification algorithm, it is recommended to select and extract good features in order to maximize the performances of the system by making task of the subsequent classification algorithm easier. Therefore it is often the case that choosing a good preprocessing and feature extraction method has more impact on the final performances than the choice of a good classification algorithm [39].
4. Classification is a step which assigns a class label to a set of features extracted from the signal. This class label corresponds to the kind of mental state identified. Classification can be performed in various ways ranging from simple thresholding or linear models to complex nonlinear neural network classifiers. The goal of classification is to assign a correct class label to a previously extracted feature vector. This class represents an intention of the BCI user. The key step for identifying neurophysiological signals in a BCI is translating the features into commands [40]. In order to achieve this step, one can use either regression algorithms or classification algorithms, the classification algorithms being by far the most used in the BCI community [41, 42].
5. Translation into a command is performed by issuing an action, corresponding to the mental state of a user identified, that is, moving the mouse cursor on a computer screen, controlling a speller, or moving a wheelchair.
6. The feedback step provides the user with information about his/her mental state. This helps the user to consciously control his/her brain activity to increase performance of the executed task.

## 4. Overview of BCI Paradigms

A variety of BCI paradigms have been exploited, such as P300 [43], SSVEP [44], ERD/ERS [45], MI [46], slow cortical potential (SCP) based [47], and hybrid methods [48–50]. We review some of these paradigms in more detail in the following subsections.

### 4.1. Spontaneous Potentials

Spontaneous EEG is measured when there is no stimulus presented to the test subject. In healthy subjects the spontaneous EEG is measured during a prolonged time span in which the brain activity changes constant waves into events with higher or lower frequency. Characteristics of different cognitive processes, mental states, and activation processes can be observed in spontaneous EEG waves. The appearance of certain frequency bands over a specific brain region can be assigned to a certain mental task. The band range limits associated with the brain rhythms, particularly beta and gamma, can be subject to contradiction and are often further subdivided into subbands that can further distinguish brain process activity with a frequency *f*, where *f* > 30 Hz or *f* < 0.5 Hz is often assumed to be of limited clinical utility; although some recent papers have published the existence of cognitive brain process in the beta, gamma, and high gamma bands [51], the literature does not clearly state whether the higher frequency activity (>30 Hz) is of cerebral origin. The EEG rhythms are affected by different actions, thoughts, and mental states. For example, the planning of a movement can block or attenuate the mu rhythm. The fact that mere thoughts affect the rhythmical activity of the brain can be used as the basis for a BCI system.

### 4.2. Event Related Potentials (ERP)

The event related brain potentials (ERP) are different from spontaneous brain activity in the way that they appear while the subject is being stimulated and are noted by performing the extensive analysis of the data. The brain generates not only uninterrupted spontaneous activity but also reacts to certain external or internal events with a characteristic potential change. On episodic stimulation, event based activity is registered, which is not displayed, if no stimulation is presented. By presenting the subject with an external stimulus (such as a click sound or a flashing light), a specific reaction and specific EEG components are expected to emerge in the ongoing EEG activity after the stimulus presentation. These ERP are analysed in the time domain using triggers, timestamps of stimulus presentation noted in the EEG. The subject is presented with a stimulus in constant intervals while his/her EEG is being recorded. The data encompassing time after the presentation of the stimulus is then analysed. The arising ERP with amplitude of 1/10 of the spontaneous brain activity is noise-like and is barely noticeable in the EEG data. After computer analysis of time samples following the stimulus and by performing averaging on the signals, the evoked potential becomes clearly visible. As most of the oscillations are not of interest, only certain frequencies are measured by selecting a time window of about 100 ms to several seconds. The observed potential has amplitude of less than 10 *μ*V and duration of around 0.5 s. It also has a typical form; after a few milliseconds of stimulus presentation, oscillations with very small amplitude arise. These potential differences are positive or negative changes in brain potential, so one can speak of cortical positivity or cortical negativity [52].

In a typical ERP, first, a small positivity is measured (called P1), followed by a negativity (called N1 or N100, appearing after approximately 100 ms) and again followed by a clear positivity (P3), which is observed after approximately 300 ms after the presentation of the stimulus, reaching its peak at about 400 ms, and known as a P300 wave [53]. The P300 and N100 waves are correlative to the stimulus and therefore observed for medical purposes; that is, in patients with multiple sclerosis, the P300 wave is often longer than in healthy patients. It also serves a purpose for diagnosing other psychological diseases such as schizophrenia, hyperactivity disorders. Apart from the sensory stimulus, ERP are evoked by other event related actions, such as imaginative or physical motorical activity, that is, the movement of arms or legs.

### 4.3. Evoked Potentials (EP)

The evoked potentials (EP) are a subset of the ERP that occur in response to or during attention to certain physical stimuli (auditory, visual, somatosensory, etc.). They can be considered to result from a reorganization of the phases of the ongoing EEG signals. The EP can have distinguishable properties related to different stimuli properties, for example, the visual evoked potential (VEP) over the visual cortex varies at the same frequency as the stimulating light [54]. Other EP such as the auditory evoked potential (AEP) are also used [55].

A distinction is made in the literature between a transient EP and a steady state EP (SSEP) based on the stimulation frequency. The former arises when the stimulation frequency is less than 2 Hz. If the stimulus repetition rate is greater than 6 Hz, a periodic response called the SSEP will result. The SSEP are defined by an increase in signal power in the band, equal to the stimulation frequency or integer multiples of that frequency. The amplitude and phase of the SSEP are highly sensitive to stimulus parameters, such as repetition rate, color contrast or sound tone, modulation depth, and spatial frequency. The SSEP was also found to be strongly dependent on spatial attention, being enlarged in the frequency of the target that has the user's attention focused on. The increased SSEP amplitudes reflect an enhancement of neural responses to a stimulus that falls within the range of spatial attention. It is this fundamental idea that justifies the use of the SSEP as a method to identify the attended target among a group of stimuli with sufficiently different stimulation rates.

There are three main modalities of stimulation:

- *Auditory One*. Signal tones of a specific frequency or clicks are used as stimuli.
- *Visual One*. Stimulus is presented as a light with a specific blinking frequency.
- *Somatosensory One*. Stimuli are elicited by electrical stimulation of peripheral nerves.

The sequence of stimulation is arranged into paradigms in order to study the responses to certain tasks. The most widely used are as follows:

- *No-Task Evoked Potentials*. The subjects are relaxed and instructed to perform no task upon stimulus reception.
- *Oddball Paradigm*. The user is requested to attend to a random sequence composed of two kinds of stimuli with one of these stimuli being less frequent than the other. If the rare stimulus is relevant to the user, its appearance triggers a P300 wave observable in the user's EEG.

### 4.4. Steady State Visually Evoked Potentials (SSVEP)

Several studies [56–58] have demonstrated an increase in neural activity excited by a visual stimulus when the test subject directs his attention to the region of visual space containing the stimulus. The results show that attention acts as a “spotlight,” enhancing the cortical representation of stimuli presented in attended regions of visual space relative to stimuli presented in the unattended regions of visual space.

Studies show that if two or more stimuli with a varying flicker frequency are presented simultaneously, neural responses are elicited by the flicker, receiving the subjects focus. The response generated by the brain corresponds in frequency to the stimulating frequency and therefore can be detected using the Fourier analysis of the EEG data. In the EEG recordings, these steady state responses are called steady state visually evoked potentials (SSVEP) [59]. If the subject directs his attention to one visual field and ignores the others while performing a target detection task, SSVEP elicited by flicker stimulation in the attended visual field have larger amplitude than SSVEP elicited by the same stimulus in trials where the other field is attended.

The use of frequency tagging to study attention has the obvious advantage of easily separating neural responses into different classes. How attention modulates the SSVEP response may depend on various parameters, such as stimulus frequency [57], stimulus spacing [60], color [61], and shape [62]. It is known that low frequency flickering induces more intensive SSVEP but might cause the users to feel uncomfortable and easily tired.

## 5. Requirements and Limitations of EEG-Based Gaming Systems

Several other factors have to be taken into account when designing a BCI system prototype. To design an end user friendly system, which could be used in everyday activities, that is, wheelchair or mouse cursor control, the system should allow its users to send commands at any time. Such a system must analyse the EEG signals continuously and determine whether the user is intending to issue control commands to the system, that is, in the control state (CS) or if he is in a no control state (NC), indicating that no control commands are issued. If the system detects the user's CS state, it must then decide which control command is being issued. We take this into account, when designing our BCI system.

The biggest problems with most BCIs are low accuracy, reliability, information transfer rate, user acceptability [63], performance variability both within and across subjects [64], and BCI illiteracy of some subjects [65]. Sometimes the output of the system does not match the input. This, of course, can be more or less serious depending on the application. If used for moving the cursor on a computer screen an erroneous output every now and then might be tolerable, but if used for controlling the motion of a physical device, such as a wheelchair, this behaviour becomes unacceptable. Another problem associated with many BCI paradigms is a long input-output delay. Today, the most successful systems work at a transfer rate of less than 30 bits per minute [66]. That might be enough to operate a simple word processor system, but it is too slow to control a wheelchair. Most research today therefore focuses on improving the two factors of speed and accuracy of BCI communication. Even though more and more BCI applications exist, there are still a number of problems BCIs needed to overcome to become interesting for the large public.

The first problem lies with the EEG sensors. Traditional EEG systems like the Biosemi ActiveTwo consist of a cap and up to 256 EEG electrodes. The high sensor count and wires make such a system impossible to use outside the laboratory, because the setup requires one or several assistants and preparation time. Another drawback is the fact that conductive gel needs to be used for leaving residue in the user's hair. The g.SAHARA system produced by g.tec does not require conductive gel, but still needs wires and an electrode cap.

BCI is often the only input modality in applications which have been developed for research projects. This can be problematic: having to control a cursor continually by means of imagined movement results in a high workload. It would probably be better if BCI was one of the multiple modalities used to control an application. Examples of such multimodal applications or hybrid BCIs are the “AlphaWoW” (Alpha-World of Warcraft) [67], where brainwaves in the alpha band are combined with keyboard and mouse inputs, and “Mind the Sheep!” [68], where SSVEP is combined with mouse input. Other examples include a touchless system [69], which combines eye gaze for cursor control with a BCI for making selections, and exergames with Brain Kinect Interface (BKI) for recording and analyzing motion capture signals and EEG signals in order to monitor motor recovery process [70].

The focus of BCI research should shift from reliability to usability and user experience [71]. This is necessary to migrate BCI systems out of the laboratory, into society. Healthy persons can choose from various alternative input modalities. So, for healthy persons to choose a BCI, the user experience and usability must be adequate. Most people have never used a BCI and the novelty of this new technology can be a reason for people to decide to use a BCI instead of alternative input modalities, even if BCI is less reliable and slower. However, if the usability is not good, people will choose a different input modality. Due to the fact that the focus in BCI research has mainly been on the reliability, no standardized methods to assess the user experience for BCI exist, yet. Several researchers (see, e.g., Kübler et al. [11]) use visual analogue scales (VAS) to rate the user satisfaction on a scale from 0 to 10. Such a rating does not provide any in-depth information about the source of satisfaction, but it allows for easy monitoring.

The final limitation to mention here is the amount of data that a BCI can transfer. The EEG measures a mixture of signals originating in neural brain activity. The two lowest functional layers of the brain are mostly locally oriented [72]. By observing brain areas responsible for these signals, it is possible to measure a corresponding signal. The higher sublevels (thoughts, emotions) are assembled on the lower levels and exist only in an abstract way. To measure physical processes on these higher layers would require additional tools to translate the measured low-level signals into the higher-level context. One needs specific “interpreters” for such operations. The problem is the interpretation of this mixture of measured signals. Hence, for the control of highly complex prostheses, EEG signals are not sufficient and can never be in the future. The signals necessary to control the arm, including the consideration of closed loop controls between the brain and the arm, ideally including the fingers and integrated touch sensors, would be too blurred to be the basis for adequate arm movement execution.

## 6. Materials and Methods

### 6.1. Hardware

The EPOC headset, designed by Emotiv Inc., has been selected as the basis for our system. The Emotiv EPOC contains 14 electrodes and 2 reference electrodes, placed in the international 10-10 system [73]; response time is 250–500 ms. The headset is designed as a video game accessory where developers are interested in using the device as a controller. The product chosen for this project was the Research Edition. This provides both the interface for programming with the headset and access to raw EEG data. The headset transmits encrypted data wirelessly. The wireless chip is proprietary and operates in the same frequency range as 802.11 (2.4 GHz). The internal sampling rate of the device is 2048 Hz. The data is then downsampled to 128 Hz before becoming available to the system for capturing the EEG signals. The captured data contains values for each of the 14 electrodes on the EPOC headset.

There are many advantages for using the Emotiv headset over other BCI and EEG devices. Many BCI devices are restrictive due to wiring. The Emotiv headset, however, is wireless and therefore offers free range of motion allowing for easy transport and setup, which is very important in an everyday use setting. Another advantage is that the EPOC does not require conductive gel for electrodes, making it easier to put on and use. Users do not have to wash their hair after using the headset. The main benefit is that it is relatively inexpensive. There are several disadvantages for the EPOC headset as well: it only uses 14 sensors, while many medical grade devices use up to four times that amount. This results in less data coming in from the brain. Additionally, more powerful devices have a sample rate of up to 1000 Hz, as opposed to the 128 Hz that EPOC runs at. Since the EPOC headset is not intended for finer signal detection, the electrodes pick up a lot of noise. Several techniques can be used to increase the Signal-to-Noise Ratio (SNR) such as band-pass filtering, averaging or class adaptive denoising [74], DCT compression [75], signal decomposition and thresholding [76], or nonlinear signal operators [77].

### 6.2. Software

The Emotiv Software Development Kit (EDK) was used for interfacing with the EPOC. It is primarily written in C, but the company also provides wrappers for accessing the Application Programming Interface (API) in C++, C#, Java, and MATLAB. MATLAB provides methods for calling functions in C code which allows for straightforward access to the EDK's API.

OpenViBE [78] is an open source graphical programming language used to design BCI applications. The aim of the OpenViBE is to provide open source software for BCI. Key features of this software are its modularity, high performance, real time data acquisition and feedback capabilities, compatibility with various hardware devices, and multiple scripting language support. It can be used to acquire, filter, process, classify, and visualize brain signals in real time.

### 6.3. Experimental Setting

The objective of the experiment was to develop a system that utilizes brain activity to offer control within a real time environment in order to evaluate signal processing algorithms. A 3-class self-paced BCI design with a NC (no control) state was chosen, as this system setup could easily be adapted for wheelchair or mouse cursor control. The system is based on the OpenViBE platform and is comprised of 5 individual scenarios, each performed in sequence. The EEG data is recorded using the Emotiv EPOC headset. Since the headset does not have any sensors over the motor cortex, obtaining even moderate results with the motor imagery (MI) approach is very unlikely. Since the sensors cover the occipital and parietal cortex reasonably, the SSVEP in the multiple visual stimuli selective attention paradigm was chosen for the experiment, due to its well-publicized success and limited subject training requirements.

### 6.4. Data Acquisition

**Figure 2:**
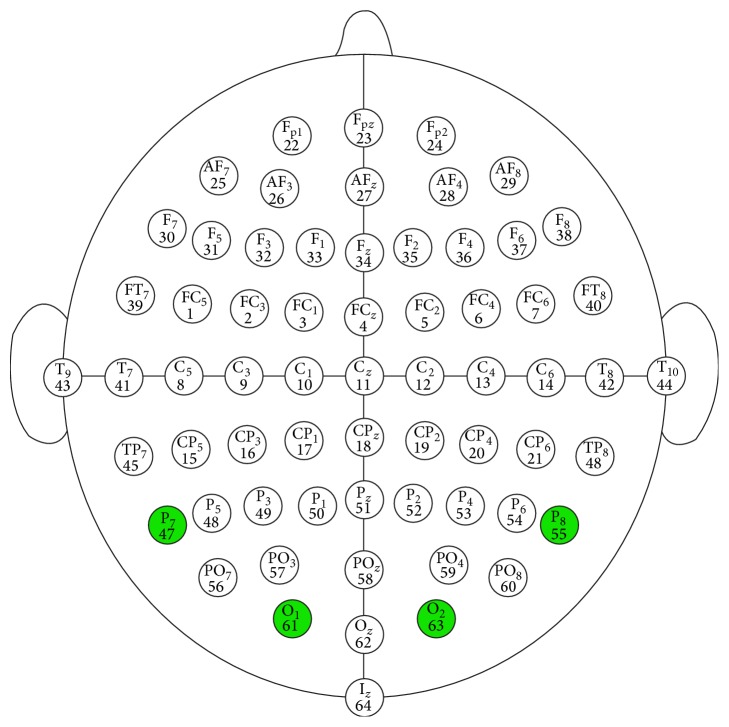
Sensor layout.

Data acquisition is performed by selecting the channels which will be used for data recording. This allows for individual sensor contact quality evaluation. The signals of interest, in the case of SSVEP, are O1, O2, P7, and P8 (see Figure 2).

**Figure 3:**
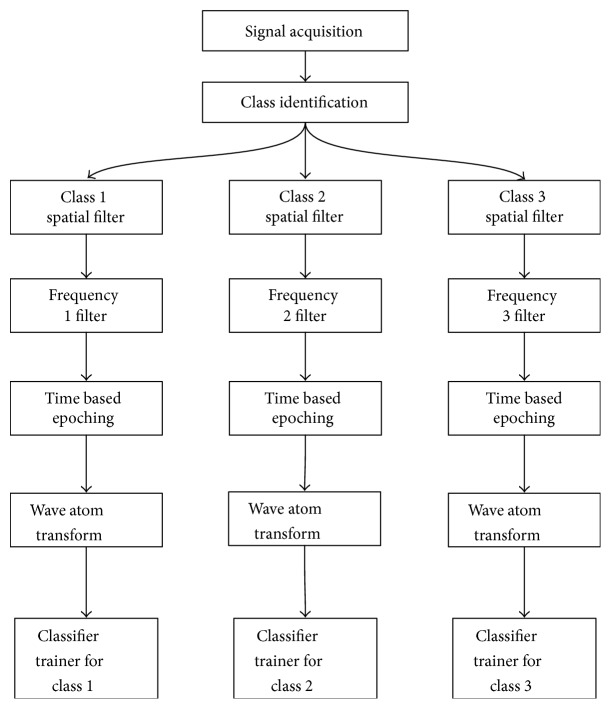
Data flow of prototype BCI shooter game.

The data is acquired in real time by the acquisition client (see Figure 3). The data is processed in the same way as the training data, in order to obtain the same feature vectors that the classifier can then identify. The output of the three classifiers is then input into a SSVEP voter algorithm, which decides on the class label of the current signal. If an NC state is detected, a class label of “0” is assigned to the trial. The control signal can then be used to move the ship and is passed to the ship control application. While in the NC state, no action is performed.

### 6.5. Data Preprocessing

The signals from the sensors are averaged and band-pass filtering of the 6–40 Hz band is performed. The signal is then split into epochs of 2 s, with a 0.5 s interval. An average signal value is obtained by averaging 4 epochs, and an FFT is then performed to visualize the different frequency bands.

Preprocessing steps are then performed in order to denoise the signal and extract relevant information features for the classifier. First, data is split into three groups, according to their corresponding class label, LEFT, RIGHT, and CENTER accordingly. This is done so that a binary classifier could then distinguish whether a trial belongs to a certain class or not, by using the “one versus all” criteria. This allows for the NC class, where output is false for all three classifiers. Next, temporal and spatial filtering is applied to each of the three groups. Specifically, each group of signals is band-pass filtered around the target frequency of interest: for the LEFT class, 29.5–30.5 Hz; CENTER, 19.5–20.5 Hz; RIGHT, 11.5–12.5 Hz. This is done, using a fourth-order Butterworth filter.

### 6.6. Feature Extraction

For feature extraction we use wave atom transform (WAT), a relatively new transform proposed by Demanet and Ying [79]. WAT performs a multiresolution analysis of a signal, that is, decomposing a signal into different frequency subbands. Wave atoms are a variant of wavelets that have sharp frequency localization and offer a sparser expansion for oscillatory functions than wavelets. Wave atoms compose wave fields as a superposition of highly anisotropic, localized, and multiscale waveforms and capture coherence of pattern across and along oscillations. WAT has been previously used mainly in image processing domain for image denoising, image watermarking, image hashing, as well as feature extraction, dimensionality reduction and numerical analysis [80], and analysis of the electrocardiogram (ECG) [81] data.

WAT is a promising approach for EEG processing because of its denoising and feature extraction capabilities and is particularly useful when the signal has discontinuities and sharp spikes as in case of EEG [82]. We expect that WAT coefficients extracted from EEG data samples can retain enough information to permit correct classification, while feature reduction should reduce network training and classification time. Wave atoms are a variant of 2D wavelet packets that retain an isotropic aspect ratio. They are well suited for representing the oscillatory patterns in a signal [80].

To extract features, we first segment the signal, extracting the 5 s long stimulation period from the trial, since only this portion of the signal carries relevant information. Next, each segment is further divided into epochs of 1 s every 0.2 s, which provides 80% overlap between neighbouring epochs. Then, WAT coefficients are obtained for every epoch, and a feature vector is aggregated. As such, 25 feature vectors are extracted for every trial. They are then used for classifier training.

As a baseline to compare WAT with, we use the band power (BP) feature method. BP performs band-pass filtering a signal in a given frequency band, then in squaring the filtered signal, and finally in averaging the obtained values over a given time window [21]. Band power features are generally computed for several frequency bands previously determined according to the mental states to be recognized. Such features have been notably used with success for MI classification [21] but also for classification of cognitive processing tasks [51]. Features are extracted by training an adaptive common spatial pattern (CSP) filter, then band-pass filtering the signal around the target frequency, as described above, and then performing band power calculation. The BP values are then used as features to train a classifier.

### 6.7. Visual User Stimulation

Visual user stimulation can be performed by using the LED or an LCD computer monitor. However, the LEDs need extra hardware to generate a constant frequency. For the purposes of this experiment, we prefer to use the LCD monitors. The drawback of using a monitor is that a stimulus frequency is limited by the refresh rate. The refresh rate should be multiple times of the stimulus frequencies; that is, for a monitor with 60 Hz refresh rate, 6.67 Hz, 7.5 Hz, 8.57 Hz, 10 Hz, 12 Hz, 15 Hz, and 20 Hz are usually used. When choosing stimulus frequencies it is also important that a frequency is not harmonic of another chosen frequency (e.g., 7.5 Hz and 15 Hz). An SSVEP response can trigger a large amplitude response not only in the main frequency, but also in the harmonic frequency, leading to missclassification. In a 60 Hz refresh rate monitor, for a 10 Hz flicker, it reverses the target colour, usually between some light and dark combination, to produce a flicker, every three frames; for 12 Hz flicker, three frames of dark followed by two frames of light colour are displayed. Therefore, the sequences of certain two frequencies could be combined to get three frequencies with a varying number of frames in each cycle (e.g., 10 Hz and 12 Hz produce 10.5 Hz, 11 Hz, and 11.5 Hz). The EPOC headset has a sampling rate of 128 Hz and therefore has low resolution at higher frequencies. The experiments using SSVEP often include stimulation frequencies of up to 60 Hz, but these should be avoided while using the EPOC. Experiments with different frequencies performed showed that best results for a three- class BCI were obtained by using 30 Hz, 20 Hz, and 12 Hz. Therefore, these frequencies were chosen for the final experimental setup.

### 6.8. Training

The training data acquisition procedure is performed several times, to obtain the training data for each of the three classes. Since the training session is time-bound by the system, it requires a lot of attention and concentration from the user and due to user fatigue has to be limited to a number of trials. In this experimental setup, a number of 20 trials per class were chosen, totalling 60 trials for a single dataset. Classifier training was achieved by gathering 4 EEG datasets of SSVEP data, acquired from 2 healthy subjects (28 years). Subjects had very few or no previous experience in BCI. During the experiments, they were asked to focus attention on targets, blinking in a defined frequency.

**Figure 4:**
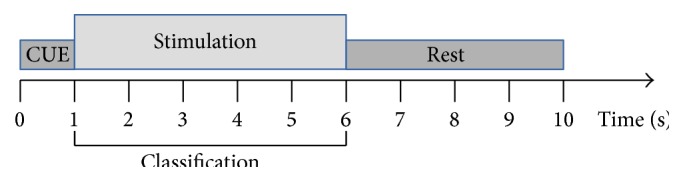
Timing of a single SSVEP trial.

A session was composed of 20 trials of each of the three classes (LEFT, RIGHT, and CENTER), arranged in a random order. The timing of the sessions was organized accordingly: in our protocol, the trial lasted 10 s. First, a yellow arrow is displayed for 1 s, indicating the target, on which the user must focus his attention. From second 1 to second 6, the trial entered stimulation phase. In this phase all three targets start blinking in their corresponding frequencies. The users are specifically ordered not to move the head, relax face muscles, and not to blink during this phase. Stimulation is then followed by a 4 s resting period, at which the user is allowed to rest his gaze, blink, or move the head. The EEG data from this period is not used for classification. This is illustrated in Figure 4.

The recorded EEG data, together with marked events, such as class labels for each trial are saved on the computer. Only relevant channels, in this case, O1, O2, P7, and P8 are used for analysis.

Since both the SVM and LDA are binary classifiers, while dealing with the three-class problem in this case (the user has to select one of the three targets), the classifiers are trained with a one-versus-all paradigm; that is, the first classifier takes features from the first frequency stimulation as the target class and stimulations from the other two frequencies as nontarget. The same is true for the second and third classifier. A voting algorithm is then used to select the class from the three classifier outputs. If all the classifier output a nontarget condition, then the state is said to be NC (no control).

### 6.9. Game Interface and Playing

**Figure 5:**
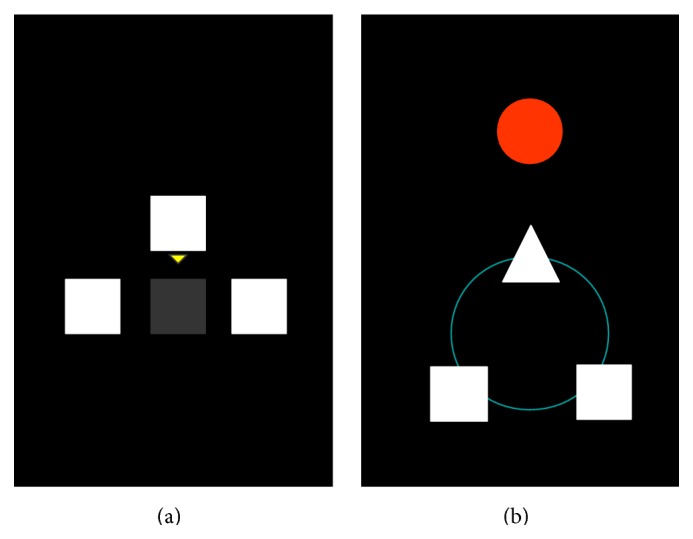
BCI game interface: training (a) and playing (b).

The colour for the flickering targets was chosen as a combination of white and black. The study [83] analysed how different colours of the targets influence classification quality. For our experiments, the white-black colour combination was chosen, since it gives the highest contrast. The user is presented with an LCD display, containing 3 blinking targets on a black background and a yellow arrow. On cue, the targets start blinking at different frequencies. This is presented in Figure 5(a).

After performing classifier training, subjects are invited to participate in a video-game-like experiment. During this game, the subjects are presented with an interface from Figure 5(b). The “spaceship” comprised two “engines,” the two rectangles, and a “cannon,” the triangle. The subject is able to rotate the spaceship by focusing his/her attention on one of the rectangular targets.

The ship is turned left or right according to the target in the user's field of attention. By focusing attention on the middle triangle, the user is able to fire the spaceship cannon. A red circular target appears next to the ship at a random location. The aim of the game is to rotate the spaceship and fire its canon to hit the red target. Once the target is hit, it disappears to reappear in another position.

**Figure 6:**
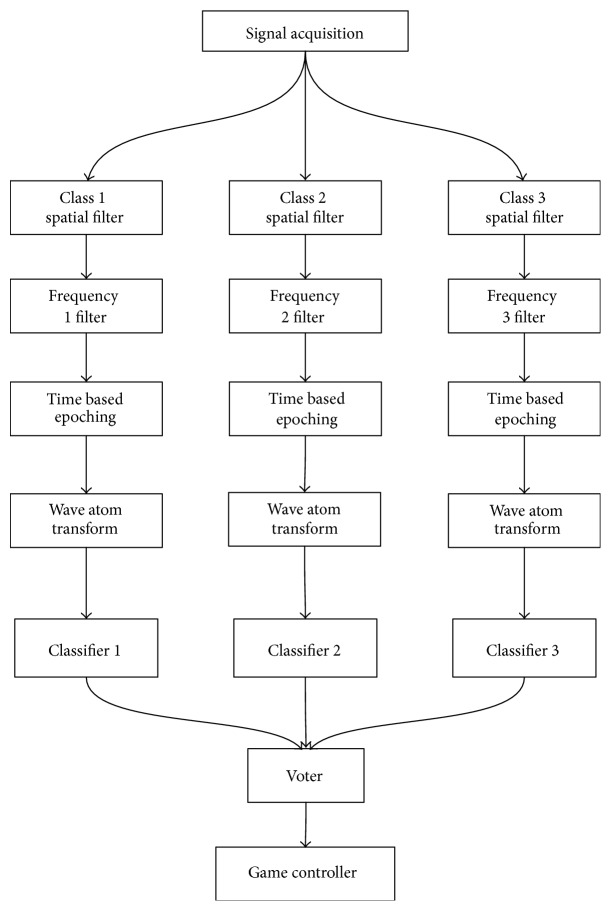
Online test shooter scenario.

The online game is executed using the OpenViBE scenario shown in Figure 6. The data is acquired in real time by the acquisition client. The data is processed in the same way as the training data, in order to obtain the same feature vectors that the classifier can then identify. The output of the three classifiers is then input into a SSVEP voter algorithm, which decides on the class label of the current signal. The control signal can then be issued to move the ship and is passed to the ship control application. If the NC state is detected, the control signal is not issued and no action is performed.

## 7. Results

To evaluate the system, two datasets have been acquired from two different subjects, marked S1 and S2. Classification has been performed using two feature extraction methods for evaluation, the wave atom transform (WAT) and band power (BP) features (here used as a baseline method to compare against). Two kinds of classifiers have been used, linear discriminant analysis (LDA) [84] and support vector machine (SVM) [85]. The SVM implementation is based on LIBSVM, available at http://www.csie.ntu.edu.tw/~cjlin/libsvm/. For the LDA classifier we used the proprietary implementation of the LDA in the OpenViBE environment. The system was implemented using the OpenViBE environment. All computations were performed on a virtual machine on a PC with Intel Core I5-3570, 3.4 GHz, 4 cores, 3.5 GB RAM.

Since there is a lack of training data, a 10-fold cross-validation is performed and accuracy is measured on the same data used for classifier training. The accuracy metric is chosen for the representation of results, since it is a simple metric that is directly linked to system usability by the user. It likely overestimates the classification result, since the classifier has been trained on the same data. The results most probably indicate higher performances than what the user will actually have during the online classification. We have performed classification using three classification methods and have compared the results.

An evaluation of the system has been conducted using two naïve subjects, named S1 and S2, unfamiliar with the BCI technology. Two feature extraction algorithms have been tested. The first algorithm used wave atom transform (WAT) coefficients. The second algorithm used the band power (BP) in the stimulation frequency band. These features were then used for classifier training. We measured the accuracy and the *F*-measure of each subject, while performing classification with 4 different classifiers (LDA, sparse LDA (sLDA), SVM with linear kernel, and SVM with RBF kernel (with parameter values *C* = 1, gamma = 10)). The results are presented in Table 1.

The results indicate that the WAT-based feature extraction method performed better than BP-based one with all four classifiers. This method can also be used in the SSVEP paradigm. Although the best results were achieved by using the SVM classifier with a linear kernel, results obtained with other classifiers are very similar. This shows that the choice of a good feature extraction algorithm is more important in BCI applications. A nonoptimal classifier can produce good results, because most models pick up on good feature data. With good features, one can use a simpler classifier that runs faster. These results also show that it is possible to develop a BCI interface system based on low-cost acquisition devices, such as the Emotiv EPOC, which performs at a reasonable usability level.

Finally, the training times (for training full dataset) for LDA and SVM classifiers are compared in Table 2. In this case, SVM outperforms LDA, too. The processing of 1 s sample of EEG data is performed in 280 ms, which allows the system to perform in real time.

The usability of the developed system was evaluated informally as the number of subjects was too small to perform formal evaluation using, for example, visual analogue scale (VAS). Both subjects complained about discomfort due to the fatigue of eyes after some time of using the system. The fatigue is caused by low frequency flickering of the game interface. The problem could be alleviated by increasing the frequency of flickering; however, it cannot be done due to the characteristics of the EPOC device. The use of a more advanced EEG equipment may solve this problem.

## 8. Conclusion

We have studied the electroencephalogram (EEG) signal processing and classification techniques in order to design brain-computer interface (BCI) systems to be used in the out-of-the-laboratory setting such as AAL environments or smart homes, with these main objectives: (1) improving efficiency in terms of accuracy of the BCI; (2) improving usability and applicability, therefore moving towards the end user; (3) designing a user friendly BCI system based on gamification principles.

We have studied the system performance while using the steady state visually evoked potential (SSVEP) paradigm. We have developed a three-class BCI system, based on SSVEP paradigm and the Emotiv EPOC headset. We have created a scenario, enabling the user to control a virtual spaceship in a game by his/her thoughts. The scenario enables the user to issue 3 control commands and has a no-command (NC) state, allowing for self-paced control. The created scenario includes classifier training, signal preprocessing, and feature extraction.

An online target shooting game, implemented in the OpenViBE environment, has been used for feedback. The wave atom transform (WAT) was chosen for feature extraction. The system achieved an average accuracy of 80.5% for both subjects, while using a support vector machine (SVM) classifier with a radial basis function (RBF) kernel. The use of WAT allowed achieving and improvement in accuracy of 4.8% when compared to the baseline band power (BP) features. These results show that BCI can be used as an interaction technique for complex applications, providing real time operation and feedback. The results also highlight that BCI can be feasible even when using low-resolution low-cost customer-grade EEG acquisition devices. This allows for reduced system cost, mobility, and subject preparation time and, consequently, allows for the subject to be prepared by a nonexpert supervisor. By improving system cost and ergonomics, the BCI technology can be used for the general public who can enjoy entertaining applications, games, and virtual reality.

Concerning the signal processing and classification part of BCI design, we believe that a better approach would be to combine, rather than selecting preprocessing, feature extraction and classification methods. Numerous methods have been proposed and tested in the BCI domain, and while some of them have sometimes been proven to perform better than others, no single method has been identified as being the best. This is partly due to the differences in system users. Therefore, we should focus on combining existing methods together and adapting them to best suit the user. Since different methods exploit different aspects of EEG, these methods could be used together in a complementary way and would probably lead to better results than when using some “single best” method alone.

In future work we plan to develop a real BCI-based game and perform experimentations on a larger number of healthy and motorically impaired subjects as well as performing usability evaluation using visual analogue scale (VAS).

## Figures and Tables

**Table 1:** Comparison of classification accuracy.

Classifier	Features	Accuracy, %		*F*1 score	
		S1	S2	S1	S2
LDA	WAT	71.5	78.2	0.64	0.67
	BP	66.2	73.2	0.56	0.62

sLDA	WAT	70.6	77.4	0.64	0.68
	BP	68.4	73.5	0.59	0.61

SVM, linear kernel	WAT	75.5	79.3	0.64	0.68
	BP	74.3	75.1	0.64	0.66

SVM, RBF kernel	WAT	**78.7**	**82.2**	0.68	0.71
	BP	74.0	77.4	0.63	0.67

S1: subject number 1, S2: subject number 2, LDA: linear discriminant analysis, sLDA: sparse LDA, SVM: support vector machine, RBF: radial basis function, WAT: wave atom transform, and BP: band power.

**Table 2:** Training time of classifiers.

Classifier	Training time, s
LDA	809
SVM	**618**
